# Hemoglobin-to-Creatinine Ratio Predicts One-Year Adverse Clinical Outcomes in ST-Elevation Myocardial Infarction: Retrospective and Propensity Score Matched Analysis

**DOI:** 10.3390/jcm14082756

**Published:** 2025-04-17

**Authors:** Luigi Spadafora, Stefano Cacciatore, Mattia Galli, Carlos Collet, Matteo Betti, Gianmarco Sarto, Beatrice Simeone, Erica Rocco, Fabrizio D’Ascenzo, Gaetano Maria De Ferrari, Ovidio De Filippo, Pierre Sabouret, Iginio Colaiori, Roberto Carnevale, Valentina Valenti, Carlo Gaudio, Francesca Romana Zimatore, Giacomo Frati, Francesco Versaci, Sebastiano Sciarretta, Giuseppe Biondi Zoccai, Marco Bernardi

**Affiliations:** 1 Department of Medical-Surgical Sciences and Biotechnologies, Sapienza University of Rome, 04100 Latina, Italy; dottormattiagalli@gmail.com (M.G.); roberto.carnevale@uniroma1.it (R.C.); valevale2012@hotmail.com (V.V.); fraticello@inwind.it (G.F.); sebastiano.sciarretta@uniroma1.it (S.S.); gbiondizoccai@gmail.com (G.B.Z.); marco.bernardi23@gmail.com (M.B.); 2 Department of Geriatrics, Orthopedics, and Rheumatology, Università Cattolica del Sacro Cuore, L.go F. Vito 1, 00168 Rome, Italy; stefano.cacciatore01@icatt.it; 3 Fondazione Policlinico Universitario “Agostino Gemelli” IRCCS, L.go A. Gemelli 8, 00168 Rome, Italy; 4 Maria Cecilia Hospital, GVM Care & Research, 48033 Cotignola, Italy; 5 Cardiovascular Center Aalst, 9300 Aalst, Belgium; carloscollet@gmail.com; 6 Department of Clinical Sciences and Community Health, Cardiovascular Section, University of Milan, 20122 Milan, Italy; matteobetti1@gmail.com; 7 ICOT Istituto Marco Pasquali, 04351 Latina, Italy; sarto.gianmarco@gmail.com (G.S.); beatricesimeone.md@gmail.com (B.S.); ericarocco.md@gmail.com (E.R.); 8 Division of Cardiology, Cardiovascular and Thoracic Department, Città della Salute e della Scienza Hospital, 10126 Turin, Italy; fabrizio.dascenzo@gmail.com (F.D.); gaetanomaria.deferrari@unito.it (G.M.D.F.); ovidio.defilippo@gmail.com (O.D.F.); 9 Heart Institute and Action Group, Pitié-Salpétrière, Sorbonne University, 75013 Paris, France; cardiology.sabouret@gmail.com; 10 National College of French Cardiologists, 75005 Paris, France; 11 UOC UTIC Emodinamica e Cardiologia, Ospedale Santa Maria Goretti, 04351 Latina, Italy; iginio.colaiori@gmail.com (I.C.); francescoversaci@yahoo.it (F.V.); 12 IRCCS NeuroMed, 86030 Pozzilli, Italy; 13 Department of Clinical, Internal Medicine, Anesthesiology and Cardiovascular Sciences, Sapienza University of Rome, Piazzale Aldo Moro, 5, 00185 Rome, Italy; carlo.gaudio@uniroma1.it; 14 Cardiovascular Diseases Residency Program, Azienda Ospedaliera Universitaria Integrata di Verona, 37126 Verona, Italy; francescazimatore@yahoo.it

**Keywords:** STEMI, myocardial infarction, anemia, renal impairment, hemoglobin to creatinine ratio

## Abstract

**Background/Objectives**: Anemia and renal impairment are key predictors of adverse outcomes in acute coronary syndromes (ACSs). The hemoglobin-to-creatinine (Hb/Cr) ratio combines these parameters into a simple index. This study aimed to evaluate its prognostic value at discharge in patients with ST-elevation myocardial infarction (STEMI). **Methods**: The primary endpoint was one-year all-cause mortality; secondary endpoints included major bleeding and the composite of all-cause mortality or reinfarction. Optimal Hb/Cr cut-off values were identified using Liu’s method. Multivariable logistic regression and propensity score matching were used to assess associations with outcomes. **Results**: We analyzed 11,236 STEMI patients from the PRAISE registry with available hemoglobin and creatinine values at discharge. The optimal cut-points were 13.68 for mortality and 14.42 for secondary endpoints. Patients were stratified into low (<13.68; 26.5%) and high (≥13.68; 73.5%) Hb/Cr groups. The low Hb/Cr group was older, had more comorbidities, and received less intensive therapy. At one year, low Hb/Cr patients had significantly higher rates of all-cause mortality (8.7% vs. 2.4%), major bleeding (5.0% vs. 2.4%), and the composite outcome (11.5% vs. 4.9%). In the multivariate logistic regression, the Hb/Cr ratio was inversely associated with all outcomes, namely all-cause mortality (odds ratio [OR] 0.94; 95% confidence interval [CI]: 0.92–0.96), major bleeding (OR 0.96; 95% CI: 0.94–0.97), and the composite endpoint (OR 0.93; 95% CI: 0.91–0.96). The Hb/Cr ratio outperformed hemoglobin and creatinine alone in predicting mortality (AUC 0.684 vs. 0.649 and 0.645; *p* < 0.001). **Conclusions**: The Hb/Cr ratio is independently associated with one-year adverse outcomes in STEMI and may serve as a simple marker of increased vulnerability. Prospective studies are needed to validate its clinical utility.

## 1. Introduction

Anemia and renal impairment are prevalent and clinically significant comorbidities among patients with acute coronary syndromes (ACSs), including ST-elevation myocardial infarction (STEMI) [1]. Anemia contributes to a myocardial oxygen supply–demand mismatch, increases cardiac workload, and promotes ventricular hypertrophy, thereby exacerbating ischemic injury in ACSs and STEMI [2]. A large systematic review and meta-analysis including 27 studies and over 230,000 ACS patients found that anemia was present in approximately 19% of cases and was independently associated with a 49% increase in the risk of all-cause mortality, persisting after over one year of follow-up [3]. Similarly, renal impairment is present in about 30% of ACS patients and contributes significantly to adverse outcomes, including in-hospital mortality, bleeding complications, heart failure, and long-term cardiovascular events [4,5,6]. Patients with chronic kidney disease (CKD) often present with traditional cardiovascular risk factors, such as diabetes, hypertension, and dyslipidemia [6]. In addition, they are affected by non-traditional, uremia-related mechanisms that further increase cardiovascular risk. These include persistent inflammation, oxidative stress, endothelial dysfunction, disruptions in calcium and phosphorus metabolism, and the accumulation of uremic toxins, all of which contribute to accelerated atherosclerosis and vascular calcification [6]. CKD also affects the metabolism and tolerability of many cardioprotective therapies, making treatment more challenging and contributing to poorer clinical outcomes in this high-risk population [7]. These conditions frequently coexist and significantly affect the prognosis of patients with ACSs [8]. In a study by Matsue et al., 53.2% had CKD, 27.8% had anemia, and anemia coexisted with CKD in 81 patients (approximately 26%) [9]. Patients with both conditions had the worst prognosis, with a multiplicative rather than additive effect on the risk of major adverse cardiac and cerebrovascular events and mortality [9]. Another study in a larger cohort of 2484 patients with coronary artery disease undergoing percutaneous coronary interventions (PCIs) found that 12.5% had both CKD and anemia, and this group showed significantly worse long-term outcomes compared to those with only one or neither condition [10].

The hemoglobin-to-creatinine (Hb/Cr) ratio has recently gained attention as a simple and cost-effective biomarker that may reflect the combined burden of anemia and renal dysfunction. This index has been evaluated in a limited number of observational studies involving patients with acute coronary syndromes (ACSs) and those undergoing transcatheter aortic valve implantation (TAVI), where lower Hb/Cr ratios have been consistently associated with increased mortality and adverse clinical outcomes [11,12,13]. In the context of patients with STEMI, the synergistic and negative prognostic impact of anemia and renal impairment is well established, with several studies incorporating these variables into clinical risk prediction models to improve outcome stratification [14,15,16,17]. Despite its potential relevance, however, the Hb/Cr ratio has never been systematically assessed in the STEMI population. Furthermore, existing data are heterogeneous, often limited to baseline measurements, and do not address the prognostic value of this index when measured at discharge.

In this study, we aimed to evaluate the association between the Hb/Cr ratio at hospital discharge and one-year clinical outcomes in patients with STEMI using data from the large, multinational PRAISE registry [18]. We explored the utility of this index as a potential surrogate marker of patient vulnerability and aimed to identify threshold values which may guide possible risk stratification strategies.

## 2. Materials and Methods

### 2.1. Study Design and Data Sources

This study is a retrospective observational analysis based on the PRAISE registry, a pooled dataset derived from several international registries and prospective cohorts of patients with ACS. The registry was originally constructed to develop and validate the PRAISE risk scores using machine learning models for the prediction of 1-year post-discharge all-cause mortality, myocardial infarction, and major bleeding following ACS [18]. It incorporates patient-level data collected from 2003 to 2019, from a total of six large-scale datasets: the BleeMACS registry (NCT02466854) [19,20], the RENAMI registry [19,20], the FRASER study (NCT02386124) [21], the Prospective Registry of Acute Coronary Syndromes in Ferrara (NCT02438085), the SECURITY randomized controlled trial [22], and the Clinical Governance in Patients with ACS project (NCT04255537). This study was conducted in accordance with the principles of the Declaration of Helsinki and received approval from the Institutional Review Boards or Ethics Committees of all participating centers involved in the contributing registries. Informed consent was obtained from all participants enrolled in the original studies whose data contributed to the PRAISE registry. Each source study followed applicable national and institutional guidelines for the ethical use of clinical data. Full inclusion and exclusion criteria and methodological details have been published previously and are available on ClinicalTrials.gov [18,19,20,21,22,23,24].

### 2.2. Study Variables

For the purposes of this analysis, a wide range of clinical, demographic, and procedural variables were extracted from the PRAISE dataset.

Baseline characteristics included patient age, sex, and medical history, including smoking status and the presence of diabetes mellitus, hypertension, dyslipidemia, peripheral artery disease, prior myocardial infarction, previous PCI, previous coronary artery bypass grafting (CABG), history of stroke or transient ischemic attack, previous major bleeding events, and malignancy.

Cardiac and renal function parameters included serum hemoglobin and creatinine levels; the estimated glomerular filtration rate (eGFR), calculated using the Modification of Diet in Renal Disease formula [25]; and left ventricular ejection fraction (LVEF).

Procedural and angiographic characteristics covered vascular access site (radial or femoral), the presence of multivessel coronary artery disease, completeness of revascularization, and the use of drug-eluting stents (DESs). Finally, detailed data on discharge pharmacological therapy were collected, including aspirin, P2Y12 inhibitors (clopidogrel, prasugrel, and ticagrelor), oral anticoagulants, angiotensin-converting enzyme (ACE) inhibitors, angiotensin II receptor blockers (ARBs), beta-blockers, statins, and proton pump inhibitors (PPIs).

### 2.3. Hemoglobin-to-Creatinine Ratio

The Hb/Cr ratio was calculated by dividing each patient’s hemoglobin concentration (g/dL) by their serum creatinine level (mg/dL), with both values measured at the time of hospital discharge. This ratio was analyzed both as a continuous variable and as a dichotomous variable based on an optimal cut-off value. The threshold used for stratification was identified through receiver operating characteristic (ROC) curve analysis, as further described in the statistical analysis section. The resulting cut-off value is specific to these units and would differ under alternative unit systems.

### 2.4. Study Endpoints

Reinfarction and bleeding events were defined according to the criteria used in the original contributing registries of the PRAISE dataset. In particular, major bleeding was classified using the Bleeding Academic Research Consortium (BARC) criteria, with type 3 (clinically relevant bleeding) and type 5 (fatal bleeding) events considered in the analysis. BARC type 3 bleeding includes overt bleeding associated with a drop in hemoglobin of ≥3 g/dL (type 3a), bleeding requiring transfusion or causing hemodynamic compromise (type 3b), or bleeding associated with cardiac tamponade or requiring surgical intervention (type 3c). Type 5 bleeding refers to fatal events, including probable (5a) and confirmed (5b) fatal bleeding, with confirmation by autopsy or imaging where applicable.

### 2.5. Statistical Analysis

The optimal threshold of the hemoglobin-to-creatinine (Hb/Cr) ratio for predicting one-year clinical outcomes was determined using Liu’s method, with bootstrapping applied to generate 95% confidence intervals (CIs) for the cut-point estimate. Sensitivity, specificity, and the area under the ROC curve (AUC) were calculated. To compare the discriminative performance of the Hb/Cr ratio with that of hemoglobin and creatinine individually, DeLong’s test was used. Although Youden’s Index suggested a slightly different cut-point, Liu’s method was selected as the primary reference due to superior classification performance. No significant differences in discrimination were observed between the two methods. Descriptive statistics were used to summarize the study population. Continuous variables were reported as mean ± standard deviation, while categorical variables were presented as absolute frequencies and percentages. Patients were stratified into low and high Hb/Cr groups according to the optimal threshold identified for predicting one-year all-cause mortality. Comparisons between groups were performed using unpaired Student’s *t*-tests for continuous variables and Pearson’s chi-square test or Fisher’s exact test for categorical variables, as appropriate. For comparisons across renal function subgroups (based on eGFR), one-way analysis of variance was used for continuous variables. To assess the association between the Hb/Cr ratio and clinical outcomes, we initially performed bivariate logistic regression, reporting odds ratios (ORs), 95% CIs, and *p*-values. Subsequently, we constructed multivariate logistic regression models adjusting for a set of potential confounders, including age, sex, diabetes mellitus, hypertension, dyslipidemia, peripheral artery disease, previous myocardial infarction, prior PCI or CABG, history of stroke, prior bleeding, malignancy, LVEF, radial access, thrombolysis, multivessel disease, use of DES, and discharge medications (aspirin, clopidogrel, prasugrel, ticagrelor, oral anticoagulants, beta-blockers, ACE inhibitors or ARBs, statins, and PPIs). To further validate our findings, we performed a propensity score-matching analysis. Patients were dichotomized into low (<13.68) and high (≥13.68) Hb/Cr groups based on the optimal cut-point for all-cause mortality. Propensity scores were estimated using logistic regression including the same covariates as the multivariable model. A 1:1 nearest-neighbor matching algorithm without replacement was applied, using a caliper width of 0.2 standard deviations of the logit of the propensity score. Covariate balance between matched groups was assessed using standardized mean differences (SMDs), with values below 0.10 considered acceptable. The average treatment effect for each outcome was calculated as the absolute risk difference between groups, and robust standard errors were used to test for statistical significance. To evaluate the incremental prognostic value of the Hb/Cr ratio over hemoglobin or creatinine alone, we conducted a net reclassification index (NRI) analysis. Predicted probabilities from logistic regression models were used to assign patients to predefined risk categories (<5%, 5–20%, and >20%), and the NRI was computed accordingly. Lastly, a sensitivity analysis compared baseline characteristics between patients with available and missing Hb/Cr values. Differences were noted in variables, such as age, comorbidity burden, and LVEF, and are addressed in detail in the limitations section. All statistical tests were two-sided, and a *p*-value < 0.05 was considered statistically significant. All statistical analyses were conducted using Stata 18.0 (StataCorp LLC, College Station, TX, USA).

## 3. Results

Of the 12,930 STEMI patients available in the PRAISE dataset, 11,236 (86.9%) had complete discharge data for both hemoglobin and creatinine and were included in the final analysis. The remaining 1694 patients (13.1%) were excluded due to missing values. As shown in Supplementary Table S1, compared to those with available Hb/Cr data, excluded patients were slightly older (62.7 ± 12.3 vs. 61.6 ± 12.7 years), more often female (25.0% vs. 21.9%), and had a lower prevalence of hypertension (38.3% vs. 51.1%), diabetes (17.0% vs. 20.6%), and multivessel disease (55.7% vs. 42.4%), but a higher prevalence of dyslipidemia (55.3% vs. 45.7%) and peripheral artery disease (10.2% vs. 4.9%), as well as a lower LVEF (47.2% vs. 50.4%).

### 3.1. Hemoglobin-to-Creatinine Ratio Cut-Off Determination

The cut-point analysis using Liu’s method for the Hb/Cr ratio in predicting one-year clinical outcomes is summarized in Table 1. The optimal threshold for all-cause mortality was identified at 13.68 (95% CI: 13.10–14.25), with an area under the curve (AUC) of 0.68. For major bleeding and the composite endpoint of all-cause mortality or reinfarction, the optimal cut-off was 14.42 (for major bleeding 95% CI: 13.90–14.94; for the composite endpoint of death or reinfarction 95% CI: 13.54–15.30), with corresponding AUCs of 0.59 and 0.60, respectively. The Hb/Cr ratio demonstrated the highest discriminative performance for all-cause mortality, with a sensitivity of 75% and specificity of 56%. A sensitivity analysis based on Youden’s Index yielded a nearly identical cut-off for all-cause mortality (13.8), with a sensitivity of 74.4%, specificity of 56.3%, and a Youden Index of 0.507. The corresponding AUC was 0.684 (95% CI: 0.656–0.712), confirming the robustness of the cut-off identified using Liu’s method.

### 3.2. Baseline Characteristics by Hemoglobin-to-Creatinine Ratio Groups

Based on the optimal discriminative value for this study’s primary endpoint (all-cause mortality), patients were stratified into low Hb/Cr (<13.68; *n* = 2982) and high Hb/Cr (≥13.68; *n* = 8254) groups (Table 2). The low Hb/Cr group was older (66.8 vs. 59.7 years, *p* < 0.001), had a higher prevalence of cardiovascular risk factors (hypertension, diabetes, and PAD, all with *p* < 0.001), and a greater burden of prior cardiovascular events, malignancy, and bleeding (all *p* < 0.001). No significant differences were found for sex (*p* = 0.069) or dyslipidemia (*p* = 0.084). The mean Hb/Cr values were 10.87 ± 2.55 and 18.62 ± 5.50 in the low and high groups, respectively. The LVEF was lower in the low Hb/Cr group (48.7% vs. 51.1%, *p* < 0.001). Procedurally, radial access and complete revascularization were more frequent in the high Hb/Cr group. In-hospital bleeding was significantly more common in the low Hb/Cr group (8.6% vs. 5.3%, *p* < 0.001), while DES use and reinfarction did not differ significantly. Considering discharge medical therapy, patients in the high Hb/Cr group were slightly more likely to be prescribed aspirin (93.9% vs. 92.7%, *p* = 0.022), although the absolute difference was minimal. Prasugrel was also more commonly used in this group (20.5% vs. 15.6%, *p* < 0.001), while ticagrelor (13.4% vs. 17.8%, *p* < 0.001) and oral anticoagulants (4.0% vs. 5.7%, *p* < 0.001) were more often administered to patients in the low Hb/Cr group. No significant difference was observed in the use of clopidogrel (65.8% vs. 65.3%, *p* = 0.603). Additionally, beta-blockers (83.8% vs. 78.5%), renin–angiotensin–aldosterone system inhibitors (ACE inhibitors or ARBs) (77.7% vs. 74.1%), and statins (95.1% vs. 91.7%) were significantly more prescribed in the high Hb/Cr group (all *p* < 0.001). At the one-year follow-up, clinical outcomes were significantly worse among patients with a low Hb/Cr ratio. Specifically, all-cause mortality occurred in 8.7% of the low Hb/Cr patients compared to 2.4% in the high Hb/Cr group (*p* < 0.001). The composite of death or reinfarction was also higher in the low Hb/Cr group (11.5% vs. 4.9%; *p* < 0.001), as well as the incidence of major bleeding (5.0% vs. 2.4%; *p* < 0.001). Baseline characteristics were examined across the stages of renal function (eGFR according to MDRD formula) and categorized according to the eGFR-based Kidney Disease Global Outcome classification (Supplementary Table S2). Patients with more advanced renal dysfunction (G3a–G5) were progressively older and showed a higher prevalence of cardiovascular risk factors and comorbidities, including diabetes, hypertension, peripheral artery disease, prior stroke, and malignancy (all *p* < 0.001). Hemoglobin and LVEF values declined with worsening renal function (both *p* < 0.001), while multivessel disease and prior bleeding events were significantly more common in the lower eGFR strata.

The logistic regression analysis demonstrated a consistent inverse association between the Hb/Cr ratio and each clinical outcome (Table 3). Lower Hb/Cr values were associated with higher odds of all-cause mortality (OR 0.87, 95% CI: 0.85–0.88, *p* < 0.001), major bleeding (OR 0.92, 95% CI 0.90–0.94, *p* < 0.001), and all-cause mortality or reinfarction (OR 0.91, 95% CI 0.89–0.92, *p* < 0.001). These associations remained significant after the adjustment for multiple clinical and procedural covariates, for all-cause mortality (OR 0.94, 95% CI: 0.92–0.96, *p* < 0.001), for major bleeding (OR 0.96, 95% CI: 0.94–0.97, *p* < 0.001), and for the composite endpoint of all-cause mortality or reinfarction (OR 0.93, 95% CI: 0.91–0.96, *p* < 0.001).

To minimize baseline differences and reduce confounding when comparing outcomes between low and high Hb/Cr groups, a propensity score-matching approach was employed using a 1:1 nearest-neighbor matching algorithm without replacement. Propensity scores were estimated through logistic regression, incorporating a broad set of clinically relevant covariates, including age, sex, hypertension, diabetes mellitus, dyslipidemia, and peripheral artery disease. The model was also adjusted for previous cardiovascular events and procedures, such as prior myocardial infarction, PCI, CABG, stroke, and major bleeding, as well as for the LVEF, radial access, use of DESs, and discharge medications (aspirin, prasugrel, ticagrelor, clopidogrel, oral anticoagulants, beta-blockers, ACE inhibitors or ARBs, and statins). After matching, 5242 patient pairs (a total of 10,484 observations) were included in the final analysis. Results showed that patients in the low Hb/Cr group had a significantly higher risk of all three clinical outcomes compared to those in the high Hb/Cr group. The average treatment effect was 2.35% for all-cause mortality (95% CI: 0.98–3.71, *p* = 0.001), 1.95% for the composite of death or reinfarction (95% CI: 0.17–3.72, *p* = 0.032), and 1.98% for major bleeding (95% CI: 0.56–3.41, *p* = 0.006) (Table 4).

Before matching, several covariates showed a substantial imbalance, with SMDs exceeding the 0.10 threshold. Post-matching, all SMDs were below 0.10, indicating a successful balancing of baseline characteristics and supporting the robustness of the adjusted outcome comparisons (Table 5).

**Figure 1:**
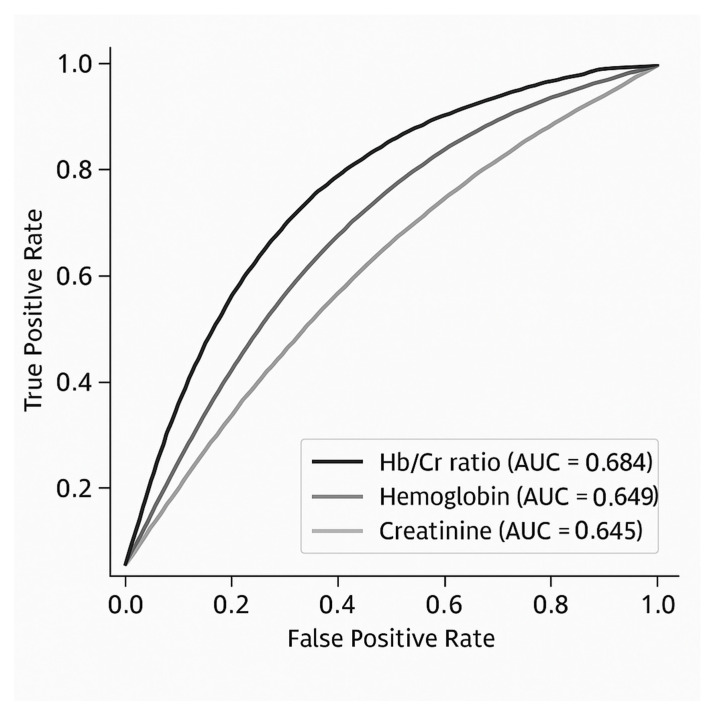
Receiver operating curves comparison between hemoglobin-to-creatinine ratio and its single components for prediction of all-cause mortality at 1-year.

To assess the added prognostic value of the Hb/Cr ratio, we compared its predictive performance for all-cause mortality with that of its individual components, i.e., hemoglobin and creatinine (Figure 1). The Hb/Cr ratio demonstrated superior discriminative ability, with an AUC of 0.684 (95% CI: 0.656–0.712), compared to 0.649 (95% CI: 0.621–0.677) for hemoglobin and 0.645 (95% CI: 0.616–0.675) for creatinine. The DeLong test confirmed a statistically significant difference among the three ROC curves (χ^2^(2) = 66.57, *p* < 0.001), supporting the incremental value of the Hb/Cr ratio over its individual components.

To further evaluate the reclassification performance, we computed the NRI using a three-tiered risk model (<5%, 5–20%, and >20% predicted mortality risk) based on predicted probabilities from the logistic regression. Compared to hemoglobin alone, the Hb/Cr ratio yielded an NRI of 5.9%, while the improvement over creatinine was even greater, with an NRI of 14.5%, suggesting a superior risk stratification performance.

**Figure 2:**
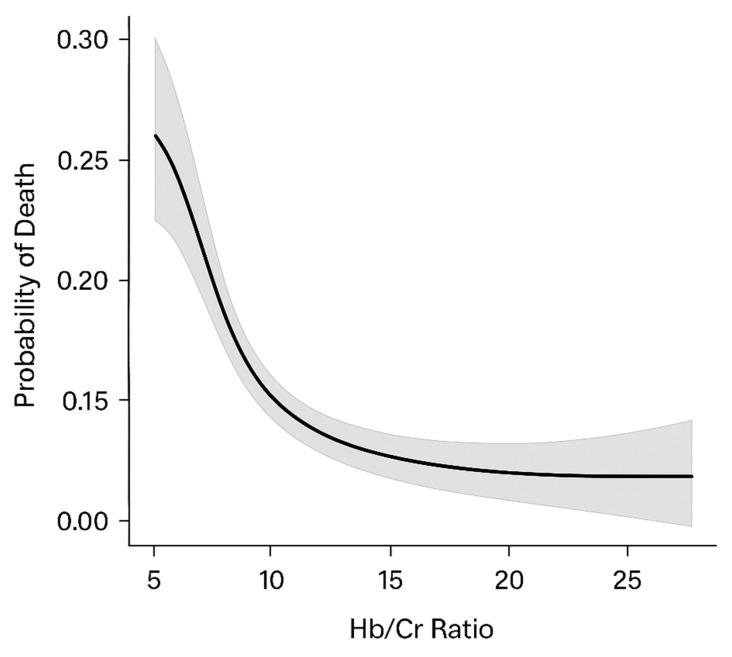
Restricted cubic spline curve showing association between hemoglobin-to-creatinine ratio and 1-year all-cause mortality.

Finally, to explore the potential non-linear relationship between the Hb/Cr ratio and mortality, we applied a restricted cubic spline logistic regression model using four knots (Table 6). This analysis revealed a significant non-linear inverse association (*p* for spline < 0.001), with the mortality risk rising steeply for Hb/Cr values below 13.0 and plateauing above 18. These findings further support the discriminative threshold of 13.68 identified by Liu’s method and highlight the marked increase in risk at lower Hb/Cr values (Figure 2).

## 4. Discussion

Our analysis provided interesting insights into the prognostic role of the Hb/Cr ratio in patients with STEMI. First, individuals with lower Hb/Cr values tended to be older, carry a greater burden of comorbidities, and were less likely to receive intensive pharmacological therapy at discharge. Second, patients with lower Hb/Cr ratios experienced significantly worse clinical outcomes at one year, including higher rates of all-cause mortality, reinfarction, and major bleeding, even after adjusting for demographic and clinical variables. Third, we observed a clear dose–response relationship, with a steep increase in the event risk for Hb/Cr values falling below 12.9. In this context, the cut-off values of 13.68 for mortality and 14.42 for major bleeding or composite outcomes emerged as the most discriminative thresholds, although these should be interpreted with caution and require external validation. Finally, the Hb/Cr ratio demonstrated a stronger discriminative ability for predicting all-cause mortality than hemoglobin or creatinine considered individually.

The impact of anemia and renal impairment on adverse outcomes in ACS is well established in the literature, with both conditions independently associated with increased mortality and complications [3,4,5,6]. This impact becomes more pronounced with greater severity and when both conditions coexist, and some studies suggest a multiplicative, rather than additive, effect on risk [8,9]. In this sense, the Hb/Cr ratio should not be interpreted as a stand-alone prognostic tool. Rather, it reflects the combined burden of anemia and renal dysfunction. Our results align with prior evidence highlighting the prognostic relevance of the Hb/Cr ratio across different cardiovascular diseases [11,12,13]. In a study by Demir et al. [12], which included 475 patients with both ST-elevation and non-ST-elevation ACS, a lower Hb/Cr ratio was independently associated with higher long-term mortality. Similarly, Numasawa et al. [11], analyzing over 150,000 non-dialysis patients undergoing PCI, reported a strong inverse relationship between the Hb/Cr ratio and in-hospital mortality and bleeding complications. These associations remained robust in multivariable models, underscoring the clinical value of this marker across large populations. In other clinical settings, the Hb/Cr ratio has also shown promise. Camci et al. [13] evaluated its role in predicting contrast-induced nephropathy in patients undergoing PCI and found it to be a significant independent predictor, with an AUC of 0.730. Ikuta et al. [26], focusing on patients undergoing TAVI, demonstrated that those in the lowest tertile of Hb/Cr values had significantly higher one-year all-cause mortality and heart failure hospitalizations. Taken together, our findings suggest that the Hb/Cr ratio may not only capture the physiological burden of anemia and renal dysfunction but also reflect a broader state of patient vulnerability. This is particularly relevant in older adults, in whom such abnormalities often coexist with a diminished physiological reserve. In this context, frailty, a clinically measurable condition characterized by increased vulnerability to stressors, is increasingly recognized as a key determinant of the prognosis in older persons with acute coronary syndromes, independent of traditional cardiovascular risk factors [27,28]. Low hemoglobin levels have been independently associated with key components of frailty, such as reduced physical activity, muscle weakness, and slowness, possibly through mechanisms like impaired oxygen delivery, tissue hypoxia, and low-grade inflammation [29]. Similarly, impaired renal function has been associated with several biological pathways contributing to frailty, including chronic inflammation, uremia, hormonal imbalance, and sarcopenia. These mechanisms, common in kidney disease, lead to a reduced muscle mass and strength, poor physical performance, and diminished resilience to stressors, ultimately promoting the development of frailty [30]. In light of these associations, it is plausible that the Hb/Cr ratio may reflect, at least in part, an underlying state of increased frailty. While our dataset did not allow for a direct assessment of frailty, future studies should investigate the relationship between Hb/Cr, established frailty measures, and adverse outcomes in older populations. In our cohort, patients with low Hb/Cr values were less likely to receive optimal discharge therapy, particularly with renin–angiotensin–aldosterone system inhibitors, beta-blockers, and statins. This pattern of less intensive treatment may partly reflect clinical caution in managing older or frailer patients, in whom comorbidities, perceived treatment intolerance, or limited life expectancy often influence therapeutic decisions. These findings echo those of Berger et al. [31], who observed that patients with ACS and CKD were less likely to receive invasive strategies or guideline-directed pharmacologic treatment. Similar concerns were noted in a systematic review by Di Mauro et al. [32], emphasizing therapeutic inertia in patients with coronary artery disease and renal dysfunction. However, this raises important questions about undertreatment in individuals who may nonetheless benefit from evidence-based therapies. While worse outcomes in our study appeared independent of discharge therapy, we must consider the substantial heterogeneity in prescribing practices within the PRAISE registry, due to its multicentric and long-term nature. Still, our findings highlight the need to avoid therapeutic inertia and to consider increased vulnerability not as a reason to reduce care intensity, but as a cue for more thoughtful, individualized treatment planning [33].

Regarding the predictive value of the Hb/Cr ratio, our analysis identified two main thresholds, namely 13.68 for all-cause mortality and 14.42 for both major bleeding and the composite outcome of mortality or reinfarction. Importantly, the risk of adverse events increased sharply when the Hb/Cr ratio fell below 12.9. These findings are in line with previous studies. Demir et al. [12] reported a mortality-associated threshold value of 14.86, while Numasawa et al. [11] identified a cut-off of 15 for predicting in-hospital mortality and bleeding risk. Defining precise cut-offs is not merely a statistical exercise; rather, it carries meaningful clinical implications. Our findings suggest that even patients with hemoglobin and creatinine levels falling within ranges typically considered only mildly abnormal may still present with an Hb/Cr ratio below 12.9, an interval associated with substantially higher risk. This underscores the importance of evaluating the combined effect of anemia and renal function, rather than assessing each parameter in isolation, when stratifying post-discharge risk in STEMI patients. Kitai et al. [34], for example, demonstrated that both mild and moderate-to-severe anemia were associated with higher cumulative rates of major adverse cardiovascular events (MACEs) in patients undergoing elective PCI, with adjusted hazard ratios showing substantial risk increases compared to non-anemic individuals. Similarly, Hussain et al. [35] found that impaired renal function, even within subclinical ranges, was independently associated with a higher risk of MACEs in younger adults.

Despite the insights provided by this analysis, there are several limitations that should be acknowledged. First, this study is based on a retrospective analysis of data from the PRAISE registry, which includes patients from multiple centers and countries over a long inclusion period (2003–2019) [18]. As such, there is inherent variability in clinical practices, myocardial infarction definitions, and treatment strategies, which may have changed substantially over time and influenced observed outcomes. Second, no external validation cohort was available to confirm the identified thresholds or associations. Furthermore, survival analyses were not performed, as time-to-event data were not collected in accordance with the original study design of the PRAISE registry [18]. Our analysis focused exclusively on one-year outcomes without incorporating longitudinal survival dynamics. Third, certain potentially relevant clinical variables, including smoking status, acute kidney injury, and contrast-induced nephropathy, were not available in the dataset and could not be accounted for in the analysis. Likewise, important contextual factors, including frailty indices, socioeconomic status, and functional status, were not recorded and may represent unmeasured sources of confounding. Fourth, approximately 13.1% of the initial cohort (1694 out of 12,930 patients) was excluded due to missing hemoglobin or creatinine values. Although comparative analyses showed only modest differences between included and excluded patients, the potential for selection bias cannot be fully excluded. Finally, although we employed rigorous statistical approaches, including multivariable logistic regression and propensity score matching, to mitigate confounding, the observational nature of this study precludes causal inference. Other unmeasured factors, such as the adherence to therapy, access to follow-up care, or center-level practices, may have influenced the outcomes observed.

In conclusion, our findings suggest that the Hb/Cr ratio may represent a simple yet informative marker of increased risk in STEMI patients, capturing the combined burden of anemia and renal dysfunction—two conditions that, even when mild, appear to synergistically worsen prognoses. The Hb/Cr ratio does not merely reflect two isolated laboratory abnormalities but may serve as a proxy for broader systemic vulnerability. Its association with adverse outcomes supports its potential utility as a complementary tool for risk stratification at discharge, particularly in identifying patients who might benefit from more intensive follow-up and therapeutic optimization. However, given the observational nature of our analysis, prospective studies are needed to confirm these findings, explore underlying mechanisms, and determine whether the Hb/Cr ratio adds incremental prognostic value beyond existing risk scores.

## 5. Conclusions

The Hb/Cr ratio at discharge is independently associated with adverse one-year outcomes in STEMI patients and may reflect the combined impact of anemia and renal dysfunction. According to our analysis, it outperforms hemoglobin and creatinine alone in predicting mortality and may serve as a simple marker of increased clinical vulnerability. While these findings are promising, the Hb/Cr ratio requires prospective validation before being considered for routine risk stratification and to determine its utility compared to currently used risk assessment tools.

## Figures and Tables

**Table 1:** Cut-point analysis for hemoglobin-to-creatinine ratio in predicting clinical outcomes at one-year follow-up.

Outcome	Hb/Cr Threshold (95% CI)	Sensitivity	Specificity	AUC
All-cause mortality	13.68 (13.10–14.25)	75%	56%	0.68
Major bleeding	14.42 (13.90–14.94)	67%	51%	0.59
All-cause mortality or reinfarction	14.42 (13.54–15.30)	68%	51%	0.60

Abbreviations: AUC, area under the curve; CI, confidence interval; and Hb/Cr, hemoglobin-to-creatinine ratio.

**Table 2:** Characteristics of participants according to hemoglobin-to-creatinine ratio.

	Low Hb/Cr Ratio (*n* = 2982)	High Hb/Cr Ratio (*n* = 8254)	*p*
*General characteristics*			
Age	66.8 (12.6)	59.661 (12.2)	<0.001
Sex, female	687 (23.0%)	1769 (21.4%)	0.069
*Medical history*			
Hypertension	1854 (62.2%)	3885 (47.1%)	<0.001
Dyslipidemia	1322 (44.3%)	3811 (46.2%)	0.084
Diabetes mellitus	906 (30.4%)	1545 (18.7%)	<0.001
Peripheral artery disease	214 (8.0%)	294 (3.8%)	<0.001
Prior myocardial infarction	400 (13.4%)	688 (8.3%)	<0.001
Prior PCI	392 (13.1%)	674 (8.2%)	<0.001
Prior CABG	55 (1.8%)	84 (1.0%)	<0.001
Prior stroke	247 (8.3%)	351 (4.3%)	<0.001
Prior bleeding	143 (5.3%)	251 (3.3%)	<0.001
Malignancy	219 (7.3%)	349 (4.2%)	<0.001
*Laboratory parameters*			
Hemoglobin, g/dL	13.1 (1.76)	14.4 (1.47)	<0.001
Creatinine, mg/dL	1.32 (0.67)	0.81 (0.15)	<0.001
HB/Cr ratio	10.9 (2.6)	18.6 (5.5)	<0.001
*Cardiac function*			
LVEF, %	48.7 (11.4)	51.1 (10.4)	<0.001
*Procedural and angiographic characteristics*			
Radial access	1074 (43.0%)	3417 (46.5%)	0.002
Multivessel disease	1244 (47.6%)	3032 (40.6%)	<0.001
DES	1401 (47.7%)	4021 (49.0%)	0.227
Complete revascularization	1436 (56.5%)	4665 (64.3%)	<0.001
Thrombolysis	59 (1.9%)	226 (2.9%)	0.036
*In-hospital events*			
In-hospital reinfarction	39 (1.4%)	97 (1.3%)	0.483
In-hospital bleeding	235 (8.6%)	412 (5.3%)	<0.001
*Discharge medical therapy*			
Aspirin	2764 (92.7%)	7750 (93.9%)	0.022
Clopidogrel	1946 (65.3%)	5430 (65.8%)	0.603
Prasugrel	465 (15.6%)	1688 (20.5%)	<0.001
Ticagrelor	532 (17.8%)	1105 (13.4%)	<0.001
Oral anticoagulants	170 (5.7%)	328 (4.0%)	<0.001
Beta-blockers	1966 (78.5%)	6119 (83.8%)	<0.001
ACE-i/ARB	1856 (74.1%)	5675 (77.7%)	<0.001
Statins	2375 (91.7%)	7101 (95.1%)	<0.001
PPi	1263 (60.5%)	3219 (52.5%)	<0.001
*Clinical outcomes at 1-year follow-up after discharge*			
All-cause mortality	258 (8.7%)	200 (2.4%)	<0.001
All-cause mortality or reinfarction	342 (11.5%)	408 (4.9%)	<0.001
Major bleeding	148 (5.0%)	201 (2.4%)	<0.001

Data are presented as absolute numbers (percentages) for categorical variables and as mean (standard deviation) for continuous variables. Abbreviations: ACE-i, angiotensin-converting enzyme inhibitor; ARB, angiotensin II receptor blocker; CABG, coronary artery bypass grafting; DES, drug-eluting stent; Hb/Cr, hemoglobin-to-creatinine ratio; LVEF, left ventricular ejection fraction; PCI, percutaneous coronary intervention; and PPI, proton pump inhibitor.

**Table 3:** Unadjusted and adjusted logistic regression models on association between hemoglobin-to-creatinine ratio and clinical outcomes at one-year follow-up after discharge.

Outcome	Unadjusted OR (95% CI)	*p*	Adjusted * OR (95% CI)	*p*
All-cause mortality	0.87 (0.85–0.88)	<0.001	0.94 (0.92–0.96)	<0.001
Major bleeding	0.91 (0.89–0.92)	<0.001	0.96 (0.94–0.97)	<0.001
All-cause mortality or reinfarction	0.92 (0.90–0.94)	<0.001	0.93 (0.91–0.96)	<0.001

* Adjusted for age, female sex, diabetes, hypertension, dyslipidemia, peripheral artery disease, radial access, drug-eluting stents positioning, cardioaspirin, prasugrel, ticagrelor, clopidogrel, oral anticoagulants, beta-blocker, angiotensin-converting enzyme inhibitor/angiotensin II receptor blocker, statin, and proton pump inhibitor. Abbreviations: CI, confidence interval and OR, odds ratio.

**Table 4:** Propensity score-matching analysis of one-year clinical outcomes according to hemoglobin-to-creatinine ratio groups.

Outcome	Matched Pairs	ATE (95% CI)	*p*
All-cause mortality	5242	2.35% (0.98–3.71%)	<0.001
Major bleeding	5242	1.95% (0.17–3.72%)	<0.001
All-cause mortality or reinfarction	5242	1.98% (0.56–3.41%)	<0.001

Abbreviations: ATE, average treatment effect and CI, confidence interval.

**Table 5:** Covariates balancing after propensity score-matching analysis.

	SMD, Before Matching	SMD, After Matching
Age	0.601	0.017
Sex	0.008	−0.040
Hypertension	0.368	0.002
Dyslipidemia	−0.054	0.003
Diabetes mellitus	0.254	−0.007
Peripheral artery disease	0.196	0.003
Prior myocardial infarction	0.116	0.013
Prior PCI	0.110	0.015
Prior CABG	0.063	−0.027
Prior stroke	0.162	−0.024
Prior bleeding	0.078	−0.024
Malignancy	0.127	−0.034
LVEF	−0.202	0.048
Radial access	−0.133	0.022
Multivessel disease	0.114	0.011
DES	−0.178	0.015
Thrombolysis	−0.075	−0.047
Aspirin	0.018	0.048
Prasugrel	−0.064	−0.048
Ticagrelor	−0.030	−0.079
Oral anticoagulants	0.080	−0.032
Beta-blockers	−0.141	0.026
ACE-i/ARB	−0.127	0.018
Statins	−0.106	0.008

Abbreviations: ACE-i, angiotensin-converting enzyme inhibitor; ARB, angiotensin II receptor blocker; CABG, coronary artery bypass grafting; DES, drug-eluting stent; LVEF, left ventricular ejection fraction; PCI, percutaneous coronary intervention; and SMD, standardized mean difference.

**Table 6:** Association between hemoglobin-to-creatinine ratio and all-cause mortality using restricted cubic spline logistic regression.

Term	Coefficient (95% CI)	SE	z-Score	*p*
Hb/Cr spline term 1	−0.214 (−0.250 to −0.178)	0.018	−11.59	<0.001
Hb/Cr spline term 2	+0.176 (+0.022 to +0.330)	0.079	+2.24	0.025
Hb/Cr spline term 3	−0.189 (−0.614 to +0.237)	0.217	−0.87	0.384
Constant	–0.254 (−0.651 to +0.142)	0.202	−1.26	0.208

The model included three spline terms (spline 1–3) representing piecewise transformations of the hemoglobin-to-creatinine ratio to capture non-linear associations, with knots placed at hemoglobin-to-creatinine values of 10, 15, 20, and 25. Abbreviations: CI, confidence interval; Hb/Cr, hemoglobin-to-creatinine ratio; and SE, standard error.

## Data Availability

Due to the different data-sharing policies of the various datasets included in this study, not all of which provided free access to data, data included in this study will not be made available. Requests for the data from each included dataset should be made to the corresponding author of each single registry and trial.

## Supplementary Materials

The following supporting information can be downloaded at: https://www.mdpi.com/article/10.3390/jcm14082756/s1, Table S1. Characteristics of participants according to hemoglobin or creatinine availability; Table S2. Baseline clinical, laboratory, and procedural characteristics of STEMI patients stratified by renal function categories according to estimated glomerular filtration rate (MDRD).
