# Supplementary material for: Hemoglobin-to-Creatinine Ratio Predicts One-Year Adverse Clinical Outcomes in ST-Elevation Myocardial Infarction: Retrospective and Propensity Score Matched Analysis
Source: J Clin Med. 2025 Apr 17;14(8):2756. doi: 10.3390/jcm14082756 (PMC12027881; doi:10.3390/jcm14082756)
Supplement: Supplementary file 1 [file jcm-14-02756-s001.zip › jcm-3483913-supplementary.pdf]

Supplementary Table S1. Characteristics of participants according to hemoglobin or creatinine availability.

	Missing Hb or Cr (n = 1,694)	Available Hb and Cr (n = 11,236)	Total (n = 12,930)
Age	62.7 ± 12.3	61.6 ± 12.7	61.7 ± 12.6
Sex, female	424 (25.0%)	2,456 (21.9%)	2,880 (22.3%)
Hypertension	649 (38.3%)	5,739 (51.1%)	6,388 (49.4%)
Dyslipidemia	936 (55.3%)	5,133 (45.7%)	6,069 (46.9%)
Diabetes mellitus	286 (16.9%)	2,315 (20.6%)	2,601 (20.1%)
Peripheral artery disease	164 (10.2%)	508 (4.9%)	672 (5.6%)
Prior myocardial infarction	160 (9.5%)	1,088 (9.7%)	1,248 (9.7%)
LVEF, %	47.2 ± 9.9	50.4 ± 10.7	50.1 ± 10.7
Multivessel disease	402 (55.7%)	4,276 (42.4%)	4,678 (43.3%)

Data are presented as absolute numbers (percentages) for categorical variables and as mean (standard deviation) for continuous variables. Abbreviations: Cr, creatinine; Hb, hemoglobin; LVEF, left ventricular ejection fraction.

Supplementary Table S2. Baseline clinical, laboratory, and procedural characteristics of STEMI patients stratified by renal function categories according to estimated glomerular filtration rate (MDRD).

	**G1 (n = 5,956)**	**G2 (n = 4,120)**	**G3a (n = 707)**	**G3b (n = 319)**	**G4 (n = 109)**	**G5 (n = 25)**	***p***
Age	58.5 ± 12.3	63.3 ± 11.8	71.1 ± 11.6	71.9 ± 11.8	71.6 ± 12.2	63.4 ± 11.6	<0.001
Sex, female	1,029 (17.3%)	946 (23.0%)	296 (41.9%)	123 (38.6%)	56 (51.4%)	6 (24.0%)	<0.001
Diabetes	980 (16.5%)	889 (21.6%)	230 (32.5%)	149 (46.7%)	55 (50.5%)	12 (48.0%)	<0.001
Hypertension	2,687 (45.1%)	2,248 (54.6%)	478 (67.6%)	228 (71.5%)	80 (73.4%)	18 (72.0%)	<0.001
Dyslipidemia	2,724 (45.7%)	1,889 (45.8%)	319 (45.1%)	135 (42.3%)	52 (47.7%)	14 (56.0%)	0.723
Peripheral artery disease	226 (4.0%)	188 (5.0%)	47 (7.8%)	30 (10.9%)	10 (11.1%)	7 (28.0%)	<0.001
Prior MI	528 (8.9%)	400 (9.7%)	102 (14.4%)	36 (11.3%)	18 (16.5%)	4 (16.0%)	<0.001
Prior PCI	526 (8.8%)	385 (9.3%)	86 (12.2%)	42 (13.2%)	25 (22.9%)	2 (8.0%)	<0.001
Prior CABG	64 (1.1%)	59 (1.4%)	12 (1.7%)	3 (0.9%)	1 (0.9%)	0 (0.0%)	0.489
Prior stroke	244 (4.1%)	227 (5.5%)	66 (9.3%)	39 (12.2%)	17 (15.6%)	5 (20.0%)	<0.001
Malignancy	248 (4.2%)	204 (5.0%)	71 (10.0%)	31 (9.7%)	10 (9.2%)	4 (16.0%)	<0.001
Prior bleeding	150 (2.9%)	175 (4.3%)	39 (5.6%)	19 (6.1%)	8 (7.5%)	3 (12.0%)	<0.001
Hemoglobin, g/dL	14.2 ± 1.6	14.2 ± 1.6	13.4 ± 1.7	12.8 ± 2.0	12.3 ± 1.8	11.3 ± 2.0	<0.001
LVEF, %	50.6 ± 10.6	50.8 ± 10.6	48.8 ± 11.6	48.0 ± 11.6	48.4 ± 12.4	46.3 ± 13.2	<0.001
Radial access	2,379 (47.3%)	1,637 (43.9%)	289 (43.0%)	132 (44.9%)	46 (46.5%)	8 (36.4%)	0.027
Thrombolysis	156 (3.0%)	105 (2.5%)	15 (2.1%)	7 (2.2%)	2 (1.8%)	0 (0.0%)	0.495
Multivessel disease	2,046 (37.3%)	1,667 (47.1%)	339 (51.7%)	167 (58.8%)	39 (40.6%)	18 (81.8%)	<0.001
eGFR, mL/min/1.73m²	115.6 ± 43.6	76.7 ± 8.4	52.9 ± 4.3	38.0 ± 4.2	24.0 ± 3.9	9.8 ± 2.7	<0.001

Data are presented as absolute numbers (percentages) for categorical variables and as mean (standard deviation) for continuous variables. Renal function categories: G1: ≥90 mL/min/1.73m²; G2: 60–89 mL/min/1.73m²; G3a: 45–59 mL/min/1.73m²; G3b: 30–44 mL/min/1.73m²; G4: 15–29 mL/min/1.73m²; G5: <15 mL/min/1.73m². Abbreviations: CABG, coronary artery bypass grafting; DES, drug-eluting stent; eGFR, estimated glomerular filtration rate; LVEF, left ventricular ejection fraction; PCI, percutaneous coronary intervention.
